# Supplementary material for: Use of open-text responses to recode categorical survey data on postpartum contraception use among women in the United States: A mixed-methods inquiry of Pregnancy Risk Assessment Monitoring System data
Source: PLoS Med. 2022 Jan 5;19(1):e1003878. doi: 10.1371/journal.pmed.1003878 (PMC8775313; doi:10.1371/journal.pmed.1003878)
Supplement: Attachment — Submitted filename: Response to Reviewers PLOSMed.docx [file pmed.1003878.s002.docx]

July 14, 2021

Dear Dr. Moyer and reviewers,

We appreciate your valuable feedback and are pleased to submit our revised manuscript “Using qualitative inquiry to improve survey data collection: An evaluation of PRAMS postpartum contraception behavior questions” for consideration in *PLOS Medicine*. We have amended figures 1 and 2, and uploaded two versions of a revised submission with and without track changes marked. Please see below for our responses to editorial and reviewer comments. We hope these responses and the subsequent changes to our manuscript address your requests. We thank you for this review of our work and look forward to a favorable response.

**Editor Requests**

1. **Abstract: Please combine the Methods and Findings sections into one section, “Methods and findings”. Methods and Findings: Please provide some additional details on the PRAMS survey, including the individuals represented and setting (e.g. the US). In the last sentence of the Abstract Methods and Findings section, please describe the main limitation(s) of the study's methodology. Conclusions: Please address the study implications both for the PRAMS questionnaire and if possible the implications for other similar surveys that may be used to evaluate postpartum pregnancy prevention use or non-use.**

We have addressed above requests to strengthen our abstract. We added “Postpartum women in the US” to better contextualize the PRAMS sample, emphasized major limitations of our qualitative inquiry, and elaborated on this paper’s contribution to contraceptive behavior measurement and the transferability of these methods for survey development.

2. **Author summary: At this stage, we ask that you include a short, non-technical Author Summary of your research to make findings accessible to a wide audience that includes both scientists and non-scientists.**

We inserted a lay summary below the abstract that includes the reason for the study, study approach and findings, and implications of these findings.

3. **As noted by Reviewer 1, the survey data seem to be relatively out of date. Please update with more recent data.**

We sincerely appreciate this request and understand that data used are from an early cycle of PRAMS phase 7. This manuscript’s primary intention is to illuminate the use of qualitative analyses in survey development using an already existing dataset. We amended our logic behind the use of these data in the methods section (lines 84 - 85). Though we acknowledge reviewer 1’s suggestion to use a more recent cycle of phase 7, we feel our current analyses convey the importance of qualitative methods in survey development as it pertains to contraceptive behavior.

4. **Methods: Thank you for including the SRQR checklist. Please add the following statement, or similar, to the Methods: "This study is reported as per the Standards for Reporting Qualitative Research (SRQR) guideline (S1 Checklist)."**

See lines 129-130.

5. **Methods: Did your study have a prospective protocol or analysis plan? Please state this (either way) early in the Methods section.**

Thank you for this question. We added a statement to clarify our traditional qualitative emerging and iterative analysis rationale used for our coding protocol (lines 106-109).

6. **Methods: Please include the relevant PRAMS questions as a supporting information file, if possible (or please indicate if these are Core Question 50 and 51 that are provided in Figure 1).**

Amended Figure 1 to indicate verbatim Core question 50 and 51.

7. **Results: It may be helpful to provide a table with relevant demographic/characteristics of the study population.**

We appreciate this suggestion and acknowledge the importance of demographics in health behavior research. Our study team discussed this manuscript’s goal as a tool for survey development rather than focusing on the behavioral data itself. We feel that including demographic information may introduce information outside of this study’s scope and misrepresent our intentions.

8. **Results: Line 118-119: Rather than referring to “numerous respondents” it would be helpful here to provide the number/percentage. Similarly, this could be changed at line 123 where “many respondents” are indicated.**

Thank you for bringing the use of strong language that may indicate a quantitative approach to our audience. We have changed wording in these cases to “sometimes, respondents”, as this portion of our findings were qualitatively emergent information and therefore not quantified. (lines 144 and 148).

9. **Discussion: Please present and organize the Discussion as follows: a short, clear summary of the article's findings; what the study adds to existing research and where and why the results may differ from previous research; strengths and limitations of the study; implications and next steps for research, clinical practice, and/or public policy; one-paragraph conclusion.**

We revised the discussion to fit these guidelines and added more detail about this study’s contribution and implications.

10. **Line 206-207: Please add the ethical approval information to the Methods section.**

Please see lines 89-90

11. **References: Please use the "Vancouver" style for reference formatting, and see our website for other reference guidelines**

References are formatted in Vancouver style and include complete citations.

**Table 1: Please make it clear in the second part of the table, that the denominators for the Non-Use reasons are 6044 for the “Raw Sample” and 6719 for the “Recoded Sample” if accurate. Please consider changing “raw” to “original” or similar, to clarify the meaning. Figure 1: Please spell out the abbreviation NFP in the figure or legend.**

We made all suggested changes to Table 1 and Figure 1.

**SRQR Checklist: Please use section and paragraph numbers, instead of page and line numbers, to refer to locations within the manuscript**

We have attached and updated SRQR checklist with appropriately labeled locations.

**Reviewer 1 Requests:**

**PRAMS is a complex survey and weights must be applied to interpret estimates. It would be nice to see how the weighted proportions of non-use differed comparing raw versus recodes responses.** **Table 1 - to me, the differences between raw and recoded estimates are not that striking for postpartum contraception use. For reasons for non-use, the proportion reporting 'other barriers' is much lower after recoding, which makes sense; but I would expect the proportions in the recoded sample to be higher compared with the raw sample (due to recoding of write-in other responses), but for most, it is lower. Why is this? Since PRAMS data should be weighted, how did the proportions compare (examining raw vs recoded) once the proportions were weighted.**

**Table 2 findings are interesting and illuminating. To put into perspective however, these responses represent a very small proportion of PRAMS respondents (<3%).**

We agree that weighted differences would be an interesting query for future studies. However, our methodological underpinnings do not support theoretical estimates. Our paper’s primary intention is to use rich individual-level qualitative data to inform survey development. We used a pragmatic approach based on previous mixed-method literature to ensure rigorous qualitative values are upheld (data richness, individual values/experiences, etc.) while also bridging the gap between qualitative and quantitative philosophy (e.g. structured coding process, kappa scores, simple recoded proportions).

**Typically, one would recode responses of "tubal ligation" as current contraceptive use (rather than non-use, at low risk for pregnancy). When you mention "abstinence" do you mean period abstinence/calendar method (which is a contraceptive method); or not being sexually active?**

We agree that sterilization procedures and abstinence should be considered contraception. We decided to keep these in the “non-use” group to show differences between original and recoded proportions. Our suggested revisions for this PRAMS question recategorizes these contraception methods as appropriate. We also suggest contraception choices that distinguish between abstaining from all sexual intercourse, abstaining from penis-in-vagina intercourse, natural family planning, and using lactation amenorrhea method.

**If you wish to look at Phase 7 because it includes more response options, at least examine the most recent Phase 7 data which is 2015.**

We appreciate this suggestion and value the importance of using recent data. Please see our response to editor comment #3 for our explanation on why these data remain valuable to our study’s intention.

**Page 3, line 77 - not all reasons for 'not using contraception' are barriers - they are often personal choice. Consider rephrasing to something that more closely matches what was measured (e.g., reasons for not using contraception at time of survey).**

Thank you for bringing this interpretation to our attention. We added definition of access barriers in our methods section to make this term clearer (lines 97-99).

**Figure 1 – 4^th^ row of boxes – change “preventing pregnancy” and “not preventing pregnancy” to “using contraception” and “not using contraception”. Contraception is not 100% effective, so using contraception does not translate to preventing pregnancy (since pregnancies can still occur).**

We appreciate and understand these concerns. We have changed some wording to reflect “using contraception” but left “preventing pregnancy” when the paper is directly reflecting the PRAMS survey questions that use the wording “not doing anything to keep from getting pregnant”.

**Reviewer 2 Requests:**

**I think authors should reflect on how the lack of proper recoding and cleaning of data may result in underestimation of contraceptive prevalence in their discussion.**

This is a great suggestion. We added the following sentence to the discussion “ A failure to capture contraception behaviors and methods like abstinence and sterilization may contribute to an underestimation of those preventing pregnancy.”

**Reviewer 3 Requests:**

**One suggestion, however, in the author's recommended reformatting, which is presented in Figure 2, is to remove the "Check all that apply" instruction from the question stem, as that ironically harkens back to the previous format, which they are recommending be revised. Indeed, the "check all that apply" instruction may encourage some respondents not to follow the yes-no response format, so I strongly recommend it be removed here.**

We appreciate your insightful feedback and have made these changes to our survey format suggestion.
