# Supplementary material for: Use of open-text responses to recode categorical survey data on postpartum contraception use among women in the United States: A mixed-methods inquiry of Pregnancy Risk Assessment Monitoring System data
Source: PLoS Med. 2022 Jan 5;19(1):e1003878. doi: 10.1371/journal.pmed.1003878 (PMC8775313; doi:10.1371/journal.pmed.1003878)
Supplement: Attachment — Submitted filename: 2Response to Reviewers PLOSMed.docx [file pmed.1003878.s003.docx]

09/16/2021

Dear Dr. Moyer and reviewers,

We again appreciate your feedback and requests. Please see our revised manuscript now entitled ““Improving pregnancy prevention behavior data collection: a mixed-method inquiry of PRAMS postpartum contraception questions” for consideration in *PLOS Medicine*. Our responses to your feedback are found below.

Best regards,

Nicole Richards

Requests from the editors:

**1. Please fully address the remaining points mentioned by Reviewer 1.**

Please see responses to Reviewer 1.
 **2. Title: Please revise your title according to PLOS Medicine's style. Your title must be nondeclarative and not a question. It should begin with main concept if possible. "Effect of" should be used only if causality can be inferred, i.e., for an RCT. Please place the study design ("A randomized controlled trial," "A retrospective study," "A modelling study," etc.) in the subtitle (ie, after a colon).**

We appreciate this comment and have changed the title to “Improving pregnancy prevention behavior data collection: a mixed-method inquiry of PRAMS postpartum contraception questions”.  **3. Abstract: Line 20-21: We suggest removing “indicating” and replacing with “potentially indicating” or “suggesting” as this is an interpretation.**

Replaced with “suggesting”.

**4. Abstract: conclusions: Line 24-25: We suggest noting “Respondents reporting contraception non-use often described in open-text responses other methods of pregnancy prevention…”
At Line 27-28, we suggest clarifying this sentence: “Our findings contribute to the understanding of how open-text survey questions can lead to more accurate depiction of postpartum contraceptive behavior.” or similar.**

We revised this portion of the abstract to a similar clarifying statement in response to Reviewer 1’s recommendation. (line 26)

**5. The Author Summary should be formatted into 3 sections (Why was this study done; What did the researchers do and find; What do these findings mean), with 2-3 bulleted points per section. Please see our author guidelines for more information:** [**https://journals.plos.org/plosmedicine/s/revising-your-manuscript#loc-author-summary**](https://journals.plos.org/plosmedicine/s/revising-your-manuscript#loc-author-summary)

We revised this section to better fit above mentioned prompts.
 **6. Introduction: The objectives presented in the final paragraph could be presented more clearly. It would be helpful if the introduction went into more detail on the significance of the study objective (such as potential for misinterpretation of questions/response options, reasons falling beyond categorical options presented; and how open-text response data may be informative in combination with quantitative data).**

We revised this section to better fit reviewer comments **7. Methods: Line 76. Please report the number of individuals included in the PRAMS survey/included in this study. Please fully describe the population and setting. Please note whether all postpartum women were mailed the questionnaires, and provide details on how a sample was selected from birth certificate data for inclusion in the PRAMS. Please note survey response rates. Please note early on whether those included are nationally representative of the population of post-partum women in the United States.**

Please see a more in-depth description of PRAMS methods on lines 92-103. **8. Methods: Line 82: Please note the year(s) of the Phase 7 PRAMS.**

Added on line 99.

**9. Methods: Line 84-89: Please provide more description of the background for this process, keeping in mind this would be intended for a broad clinical/medical audience.**

We have addressed this through adding to the conceptual framework and study protocol detail (lines 108-117) **10. Methods: Original data analysis: Lines 91-103: Please mention here where the open-text responses are incorporated/evaluated, including the numbers of qualitative responses.**

We appreciate this comment and have inserted more detail about this step in lines 113-114.

**11. Methods: Line 112: Please note if the “PRAMS examples” indicates the categorical response options provided.**

We clarified that these were examples given to respondents in core question 50.

**12. Methods: Please note in the Methods if your study had a prospectively developed analysis plan. Did your study have a prospective protocol or analysis plan? Please state this (either way) early in the Methods section.
a) If a prospective analysis plan (from your funding proposal, IRB or other ethics committee submission, study protocol, or other planning document written before analyzing the data) was used in designing the study, please include the relevant prospectively written document with your revised manuscript as a Supporting Information file to be published alongside your study, and cite it in the Methods section. A legend for this file should be included at the end of your manuscript.
b) If no such document exists, please make sure that the Methods section transparently describes when analyses were planned, and when/why any data-driven changes to analyses took place.
c) In either case, changes in the analysis-- including those made in response to peer review comments-- should be identified as such in the Methods section of the paper, with rationale**

We explain our recategorization protocol in more depth within our conceptual framework elaboration on lines 108-117. We discuss how this protocol was used in lines 134-150. **13. Methods: Qualitative analysis: Please completely describe the conceptual framework underlying your qualitative analysis. It would be helpful to provide more description on this process, keeping in mind a broad, general audience.**

We elaborated on how quantitative and qualitative epistemologies were used in each stage of our analyses, highlighting where subjective qualitative accounts were merged with objective quantitative approaches to manage interpretation bias. (lines 108-117)

**14. Results: Line 133: It is slightly confusing to refer to this as “original sample” and could be renamed “original response data” or similar.**

We renamed original sample to original response data. **15. Results: Please mention the total numbers included in the sample. Please present numerators and denominators for percentages.**

We added the numerator and denominator for original response data on line 170. **16. Results: Line 140: Please clarify if the 30% reporting additional reasons indicates open-text responses.**
We now include “via open-text responses” at the end of this sentence.

**17. Results: Line 153: Please provide the number of responses that were recoded based on the open-text responses.**

Via our qualitative recategorization methods, we did not collect this information. Some respondents were moved from using contraception to non-use categories and vice versa. Instead, we have included the net number of women added to non-use category (675). **18. Discussion: The organization of the discussion could be further refined. Please present and organize the Discussion as follows: a short, clear summary of the article's findings; what the study adds to existing research and where and why the results may differ from previous research; strengths and limitations of the study; implications and next steps for research, clinical practice, and/or public policy; one-paragraph conclusion.
Please begin with a statement reflecting the main findings of the study, rather than beginning with interpretations (“...illuminated an unclear understanding…”). We suggest expanding on the discussion of existing literature, and limitations of the study.**

We reorganized the discussion and discussed study limitations. We also cited literature of general ways qualitative inquiry is used in open-text data and explain how our mixed-method approach adapts that to a survey improvement tool.

**19. Line 186: Please clarify the sentence describing “respondents missing skip patterns”**

Line 224 explains participant confusion surrounding skip patterns. **20. Line 236: Please move this statement to the Methods, including the exemption number.**

Moved to lines 117-118.

**21. Line 238: Please remove the Consent for Publication statement.**

Removed.
 **22. Line 239: Please remove this section, and ensure all information on data availability are included in the manuscript submission metadata.**

Removed.

**23. Lines 242-249: Please remove the Competing Interests, Funding, and Author contributions sections from the main text, and check that this information is complete and accurate in the relevant sections of the manuscript submission metadata.**
Removed.

**24. Table 2: Please include the definition of all abbreviations in the legend (LAM).**

Included and indicated with an asterisk.

**25. Figure 2: Please define the abbreviation SP.**

We removed the abbreviation and replaced it with “Survey Participant”

**Comments from the reviewers:**

**Reviewer #1: * PRAMS data are population-based but not nationally representative (although they represent a large proportion of US births). The text (e.g., abstract) still refers to PRAMS as being nationally representative which is incorrect.**

We changed our wording to “national data” rather than “nationally representative” and omitted the use of “representative” throughout the manuscript.

*** PRAMS Phase 7 was conducted 2012-2015; PRAMS Phase 8 began in 2016 and 2019 data are now publicly available with 2020 data expected in the coming months. I can understand why the authors do not wish to replicate the work using Phase 8 2019 data given this would be very time intensive, but without doing so, I'm not sure how informative the report is related to the last sentence of the abstract: "This PRAMS question can be revised to improve clarity, including a more comprehensive list of pregnancy prevention methods and non-use options." Perhaps drop that sentence and make the conclusions a bit more broad - something along the lines of how examining write-in response can improve survey quality to inform both PRAMS and other similar surveys.**

We appreciate this comment and have broadened our concluding abstract statement as requested.

*** I do not agree with the author's response to not present weighted estimates of contraceptive non-use. Estimates of PRAMS data should always be weighted so the estimates are population-based. Of course the in-depth examination of write-in responses is done at the individual level, but for one to meaningfully compare how different or similar estimates of non-use are comparing original/raw vs recoded data, those estimates should be weighted. Otherwise the reader cannot tell if the recoding had a meaningful impact on proportions.**

We added the weighted percentages to Table 1.

**New sentence added to the discussion in response to Reviewer #2 seems a bit off to the reviewer's intent. Rather than "A failure to capture contraception behaviors and methods like abstinence and sterilization may contribute to an underestimate of those preventing pregnancy"….I suggest framing it around the importance of data cleaning/recoding and examining write-in response to improve data quality/validity. Because in reality, PRAMS will not be able to add new response options. To add a new response option, another one must come off given space limitations on the survey (PRAMS is hard copy vs BRFSS which is phone-administered and not every response is read by the interviewer). PRAMS questions deemed to have too many possible response options to be valid might instead be removed from the survey altogether, which I believe would be an unintended consequence of the paper. So careful framing is needed. The data aren't junk - analysts should just carefully examine write-in responses and recode appropriately.**

This is a very good point. This question may look differently based on a survey’s collection method and overall mission. We agree that this PRAMS question may not take precedence over the abundant important information collected from postpartum individuals. We have added this in the discussion as another consideration and removed the sentence mentioned above.
