# Supplementary material for: Use of open-text responses to recode categorical survey data on postpartum contraception use among women in the United States: A mixed-methods inquiry of Pregnancy Risk Assessment Monitoring System data
Source: PLoS Med. 2022 Jan 5;19(1):e1003878. doi: 10.1371/journal.pmed.1003878 (PMC8775313; doi:10.1371/journal.pmed.1003878)
Supplement: Attachment — Submitted filename: 3Author Response 111621.docx [file pmed.1003878.s004.docx]

November 17, 2021

Dear Dr. Moyer and reviewers,

We are pleased to submit our revisions for our manuscript now entitled “Use of open-text responses to recode categorical survey data on postpartum contraception use among women in the United States: A mixed-methods inquiry of Pregnancy Risk Assessment Monitoring System data" Our responses to your feedback are found below.

Best regards,

Nicole Richards

**Requests from Editors:**

**1. Please address the points of Reviewer 1, including avoiding referring to the PRAMS survey as national (e.g at line 4, 67, and throughout) and tempering conclusions to reflect that original and re-coded findings lead to similar estimates from a clinical perspective, but noting that examination of responses could be useful for improving data quality.**

We appreciate these points and have addressed them throughout the manuscript (see specific responses below).

**2. Title: We suggest revising to "Use of open-text responses to recode categorical survey data on postpartum contraception use among women in the United States: A mixed-methods inquiry of Pregnancy Risk Assessment Monitoring System data" or similar, but please include "United States" in the title.**

We accept this title and have changed it in our resubmission.

**3. Abstract: Line 4: Please change this to “Pregnancy Risk Assessment Monitoring System (PRAMS).**
Thank you for bringing this to our attention. We added the missing word “assessment”.

**4. Abstract: Line 24-25: We suggest revising the sentence to: “In this study, we observed that respondents reporting contraception non-use…” or similar.**

We appreciate this suggestion and have made this change.

**5. Author Summary: Please organize the author summary into sets of 2-3 single sentence bullet points for each of the following questions. For an example, please see:** [**https://doi.org/10.1371/journal.pmed.1002416**](https://doi.org/10.1371/journal.pmed.1002416)
 **6. Author summary: Please revise the following bullet points:

-”We used qualitative methods to uncover richer information about respondents’ postpartum contraception behavior and interpretations of the survey question” -please remove “richer” or replace with “more detailed information” or similar.
-We suggest revising the next point to: “We observed that qualitative responses added to and altered quantitative contraception behavior reports, and that reasons for contraception non-use are incompletely captured by existing response categories.” or similar.
-We suggest revising this point to: “Family planning professionals may be able to obtain significant and detailed information about contraceptive behavior from open-ended responses, and this information may be useful to adjust clinical approaches to postpartum contraception conversations.” or similar.**

We have incorporated your suggestions and provided more detail per the author summary content instructions. (lines 36 – 58)

**7. Introduction: Line 67-68: Please refer to PRAMS as a population-based rather than national surveillance system.**

Changed to “population-based”. (line 77-78)

**8. Introduction: Line 75-77: Please revise to: “The objective of these analyses was to identify how responses to PRAMS survey questions match respondents’ consistent and accurate (reliability and validity) interpretations through the additional analysis of qualitative observations.” or similar, to clarify.**

Revised to your suggestion. (lines 85 – 88)

**9. Methods: Line 79: Please revise the following to provide more relevant information on the PRAMS project (**[**https://www.cdc.gov/prams/index.htm**](https://www.cdc.gov/prams/index.htm)**):

“PRAMS, a surveillance project run by the Centers for Disease Control and Prevention (CDC) and state and local health departments, collects state birth certificate data and includes both universal core questions and site-specific questions about maternal attitudes and behaviors related to pregnancy [reference].” or similar.

Please mention the fact that PRAMS covers approximately 81% of births in the United States (or please cite the most accurate estimate from the CDC).**

Suggested change made and altered to include percent of US births on lines 91 – 94.

**10. Methods: Line 80: Please provide more details on how postpartum women were selected to be mailed the questionnaires (e.g. it seems as if questionnaires are sent 2-4 months after delivery, to a sample drawn monthly from current birth certificate data).**

Lines 95-97: This information was added “Postpartum women are identified through birth certificate data and mailed questionnaires 2-4 months after delivery, and. Potential participants are followed up via mail and phone to encourage higher participant response rates.”

**11. Methods: Line 86: In the abstract, PRAMS 2012 data are mentioned, and here a range of 2012-2015 is mentioned. Please clarify the year during which survey data were collected for this analysis.**

Lines 102, 106-108: We added “a subset of phase seven…” and moved the sentence “We chose only the 2012 cycle as a standard sample that could both satisfy quantitative value and reach qualitative saturation” to this paragraph to better communicate our subset analysis.

**12. Methods: Line 110: Please check if this should be “...postpartum contraception uptake barriers…”**

We added “contraception” to clarify our statement. (line 129)

**13. Methods: Line 117: We suggest noting that “Original postpartum contraception use prevalence data were analyzed using SPSS software version 26 (IBM).” or similar. Please similarly clarify this for the mention of Excel (Line 119), Stata (Line 145) and NVivo (Line 148).**

We removed previous information in parentheses and inserted all software names and versions into our sentences instead. (lines: 136-137, 163, 166)

**14. Results: Line 181: Please remove “...suggesting the importance of recoding.” as this is an interpretation.**

Thank you. This statement was removed.

**15. Discussion: As Reviewer 1 points out, please temper conclusions to mention and reflect on the fact that the original and recoded findings are significantly different statistically, but are similar, in terms of clinical implications but that the examination of responses could be useful for improving data quality.**

Lines 228-230: We’ve added a follow up sentence to our discussion surrounding this point: “Indeed, while several overall weighted percentages in each category were the same or only different by tenths of a percent, chi-square tests indicate that the recoded categories were all statistically significantly different after this recoding exercise. *That is, our original and recoded findings yield similar measures of contraception use, but statistical significance shows the value of re-examining survey responses to improve data quality*.”

**16. Discussion: Line 215: We suggest revising to: “...****this suggests that additional categorical options could be useful to capture a more comprehensive picture of contraception behavior.”**

Revised on lines 236-237.

**17. Discussion: Line 218: We suggest removing the word “national” and replacing with “population-based” or similar.
18. Discussion: Line 234: Please avoid the word “national” and replace with “population-based” or similar.**
All “national” references were replaced with “population-based”

**19. Discussion: Line 260: Please revise to “may lead to” in the sentence.**

Revised on line 282

**20. References: Please provide the complete citation information for ref 14.**

Citation is now complete, including DOI.

**21. Table 1: Please do not report "p<0.05" and instead provide exact p values for each analysis in the weighted % column (including p values for results not reaching statistical significance).**

While we appreciate this suggestion, all p values were 0.000, perhaps due to the large sample size. We have changed the legend to “p value = 0.000” instead of “p value <0.05”

**22. Figure 1: There seem to be two versions of this figure. Please include a descriptive legend in addition to the title, for this figure. Please indicate natural family planning (NFP) in the legend.
23. Figure 2: Please include a descriptive legend in addition to the title for this figure. Please define all abbreviations used in the legend (IUD).**

We have inserted descriptive legends, including defined abbreviations.

**24. SRQR Checklist: Please refer to Methods, paragraph 2 as the location for IRB/ethical review information. Please also refer to Competing Interests and Financial Disclosures sections as locations for Conflict of Interest and Funding information.**

Thank you. We have made these changes.


**Comments from Reviewers:**

**Reviewer #1: PRAMS data are population-based but not national (although they represent a large proportion of US births); data are state/site-specific not national. Abstract line 4 still refers to PRAMS as being national.**

We have changed the phrasing to “population-based” as requested.

**I appreciate the authors comparing weighted proportions of original vs recoded variables; however, although statistically different via chi-square tests (page 8, lines 208-209), the differences are not meaningful from a clinical or public health surveillance perspective. When I look at Table 1 - I feel reassured that both methods (original vs recoded) produce very similar estimates of postpartum contraception use (79% vs 77%). That's an OK conclusion to have as well; you could still encourage analysts to examine write-ins (time permitting) to improve data quality.**

We appreciate these comments and have implemented your suggestion in the discussion.
